# Genome Sequences of Multiresistant *Staphylococcus capitis* Pulsotype NRCS-A and Methicillin-Susceptible *S. capitis* Pulsotype NRCS-C

**DOI:** 10.1128/genomeA.00541-16

**Published:** 2016-06-09

**Authors:** H. Lemriss, Y. Dumont, S. Lemriss, P. Martins-Simoes, M. Butin, L. Lahlou, J. P. Rasigade, S. El Kabbaj, F. Laurent, A. Ibrahimi

**Affiliations:** a Department of Biosecurity PCL3, Research and Medical Analysis Laboratory of Gendarmerie Royale, Rabat, Morocco; b International Centre for Research in Infectious Diseases, INSERM U1111, University of Lyon, Lyon, France; c Department of Clinical Microbiology, Northern Hospital Group, Hospices Civils de Lyon, Lyon, France; d Biotechnology Laboratory (Medbiotech), Medical and Pharmacy School, University Mohammed V de Rabat, Rabat, Morocco; e National Reference Center for Staphylococci, Hospices Civils de Lyon, Lyon, France

## Abstract

Here, we report the draft genome sequences of methicillin-susceptible *Staphylococcus captis* pulsotype NCRS-C (CR02 strain) and multiresistant *Staphylococcus captis* pulsotype NCRS-A (CR07 strain).

## GENOME ANNOUNCEMENT

*Staphylococcus capitis* is a Gram-positive coccus belonging to the coagulase-negative staphylococcus group (CoNS) that is frequently found on the human skin and mucosa (1). Recently, a few studies have reported the emergence of *S. capitis*, which was found to be a major cause of late-onset sepsis (LOS) in several neonatal intensive care units (NICUs) (2, 3). A clonal population of methicillin-resistant *S. capitis* NRCS-A strains has spread into several geographically distant NICUs (4). These isolates exhibit reduced susceptibility to vancomycin, which is the most widely used antimicrobial agent in the NICU setting (5, 6).

In order to elucidate the molecular mechanisms behind the wide-spreading of methicillin-resistant *S. capitis* and methicillin-susceptible *S. capitis* in NICUs in France, we sequenced two different pulsotypes (NRCS-A and NRCS-C) of *S. capitis* strains (CR07 and CR02).

In this report, we present the draft genome sequences of multiresistant *S. capitis* pulsotype NRCS-A and methicillin-susceptible *S. capitis* pulsotype NRCS-C isolated from blood cultures from NICUs in Lyon, France.

These two pulsotype strains were grown in blood agar at 37°C, and genomic DNA was extracted using the PureLink genomic DNA kit (Invitrogen), according to the manufacturer’s recommended protocol. The DNA libraries were prepared from 1 µg DNA genomic extracted following GS rapid library protocol (Roche 454; Roche).

The genome sequence of each *S. capitis* strain was determined by high-throughput sequencing performed on a Genome Sequencer FLX + system (454 Life Sciences/Roche) using FLX Titanium reagents, according to the manufacturer’s protocols and instructions. *De novo* assemblies were performed using Roche Newbler assembly software (version 2.9).

An automatic syntactic and functional annotation of the draft genome was performed using the MicroScope platform pipeline (7). The syntactic analysis combines a set of programs including AMIGene (8), tRNAscan-SE (9), RNAmmer (10), Rfam scan (11), and Prodigal software (12) to predict genomic objects that are mainly coding sequences (CDSs) and RNA genes. More than 20 bioinformatics methods were used for functional and relational analyses. The homology search was performed in the generalist databank UniProt (13) and in more specialized databases such as COG (14), InterPro (15), PRIAM profiles for enzymatic classification (16), prediction of protein localization using TMHMM (17), SignalP (18), and PsortB (19) tools.

The draft genome sequence of methicillin-susceptible *S. capitis* NRCS-C (CR02 strain) has 33.05% G+C content, is 2,344,750 bp in length which is distributed in 320 contigs (average coverage, 7.0×) with 2,415 genes, 2,476 CDSs, 42 pseudo genes, 4 rRNAs (5S,16S, 23S), and 59 tRNAs.

The draft genome sequence of methicillin-resistant *S. capitis* NRCS-A (CR07 strain) has 32.83% G+C content, is 2,477,101 bp in length which is distributed in 26 contigs (average coverage, 31×) with 2,411 CDSs, 4 rRNAs (5S,16S, 23S), and 61 tRNAs.

### Nucleotide sequence accession numbers.

These whole-genome sequences were deposited at GenBank under the accession numbers CZWI00000000 and CZWH00000000 for a methicillin-susceptible *S. capitis* NRCS-C strain (CR02) and a multiresistant *S. capitis* NRCS-A strain (CR07), respectively. The versions described in this paper are the first versions.
